# Synergistic inhibition of glioblastoma multiforme through an *in-silico* analysis of luteolin and ferulic acid derived from *Angelica sinensis* and *Cannabis sativa*: Advancements in computational therapeutics

**DOI:** 10.1371/journal.pone.0293666

**Published:** 2023-11-09

**Authors:** Mohd Suhail, Mohammad Tarique, Shams Tabrez, Torki A. Zughaibi, Mohd Rehan

**Affiliations:** 1 King Fahd Medical Research Center, King Abdulaziz University, Jeddah, Saudi Arabia; 2 Department of Medical Laboratory Sciences, Faculty of Applied Medical Sciences, King Abdulaziz University, Jeddah, Saudi Arabia; 3 Department of Child Health, School of Medicine, University of Missouri, Columbia, Missouri, United States of America; Gauhati University, INDIA

## Abstract

The primary objective of this study is to uncover novel therapeutic agents for the treatment of Glioblastoma Multiforme (GBM), a highly aggressive form of brain cancer, and Alzheimer’s Disease (AD). Given the complexity and resistance associated with both conditions, the study underscores the imperative need for therapeutic alternatives that can traverse the biological intricacies inherent in both neuro-oncological and neurodegenerative disorders. To achieve this, a meticulous, target-based virtual screening was employed on an ensemble of 50 flavonoids and polyphenol derivatives primarily derived from plant sources. The screening focused predominantly on molecular targets pertinent to GBM but also evaluated the potential overlap with neural pathways involved in AD. The study utilized molecular docking and Molecular Dynamic (MD) simulation techniques to analyze the interaction of these compounds with a key biological target, protein tyrosine phosphatase receptor-type Z (PTPRZ). Out of the 50 compounds examined, 10 met our stringent criteria for binding affinity and specificity. Subsequently, the highest value of binding energy was observed for the synergistic binding of luteolin and ferulic acid with the value of -10.5 kcal/mol. Both compounds exhibited inherent neuroprotective properties and demonstrated significant potential as pathway inhibitors in GBM as well as molecular modulators in AD. Drawing upon advanced *in-silico* cytotoxicity predictions and sophisticated molecular modeling techniques, this study casts a spotlight on the therapeutic capabilities of polyphenols against GBM. Furthermore, our findings suggest that leveraging these compounds could catalyze a much-needed paradigm shift towards more integrative therapeutic approaches that span the breadth of both neuro-oncology and neurodegenerative diseases. The identification of cross-therapeutic potential in flavonoids and polyphenols could drastically broaden the scope of treatment modalities against both fatal diseases.

## 1. Introduction

Glioblastoma Multiforme (GBM), an aggressively invasive astrocytic neoplasm, is recognized for its angiogenic propensities, pronounced vascular expansion, and predilection for necrotic transformation [1]. Despite extensive research efforts, its clinical trajectory remains a significant concern due to the limited efficacy of conventional therapies. The contemporary landscape places GBM as a particularly daunting cerebral neoplasm, characterized by its recalcitrant nature and concomitantly truncated survival rates [2–4]. The World Health Organization’s classification system delineates astrocytomas on a continuum anchored by an amalgam of genetic attributes, histopathological nuances, and determinants of malignancy [5]. This spectrum ranges from the benign presentations, such as oligodendrogliomas, to formidable glioblastomas [5]. Although benign variants are often more manageable, malignant gliomas are unusual with remarkable genetic complexity and associated malignancy delineations. A salient observation in the GBM paradigm is the histopathological congruence between the primary and secondary subtypes, despite their disparate genetic origins. Within this context, giant cell glioblastoma has emerged as a distinct subtype, typified by its conspicuous multinucleated cellular configuration [6]. The elucidation of IDH mutations as seminal in glioma pathogenesis not only underscores their role within this framework but also extrapolates their significance across diverse malignancies [7]. From a diagnostic perspective, salient indicators such as overt necrosis and MVP patterns have been underscored as pivotal for diagnostic and prognostic stratifications in GBM [8]. A rigorous investigation into these molecular imprints could provide a more nuanced understanding of glioma ontogenesis, driving enhanced prognostic capabilities and facilitating the genesis of tailored therapeutic paradigms [9].

GBMs, in their clinical manifestations, are characterized by insidious growth trajectories, precipitating escalated intracranial pressures. This, in turn, manifests as a spectrum of neurological perturbations, encapsulating visual disruptions, exacerbated cephalalgias, and marked cognitive aberrations. Concurrently, at the molecular interface, recent studies have highlighted the contributory role of single nucleotide polymorphisms in an array of conditions, traversing from GBM to neurodegenerative entities such as Alzheimer’s [10–13]. Cutting-edge findings illuminate the association between amyloid-beta (Aβ) peptide and glioma cellular phenotypes, with subsequent histochemical analyses affirming amyloid deposition in human glioma derivatives [14]. Amidst this intricate landscape, phytochemicals have re-emerged as potential therapeutic agents, with compounds such as ferulic acid gaining prominence for their anticancer properties [15]. Our study is uniquely positioned at this intersection and aims to evaluate the synergistic effects of luteolin and ferulic acid in modulating GBM outcomes.

Luteolin and ferulic acid are natural compounds with well-established medicinal properties, demonstrating significant potential for synergistic inhibition of Glioblastoma Multiforme (GBM), a highly aggressive brain tumor. Luteolin, found in foods like celery and chamomile, has garnered attention in cancer research due to its antioxidant and anti-inflammatory properties, which have shown promise in inhibiting cancer cell growth and promoting apoptosis [16–18]. Furthermore, the ability of luteolin to modulate signaling pathways involved in cancer progression makes it an attractive candidate for cancer therapy. Ferulic acid is abundant in whole grains and certain fruits, and possesses potent antioxidant properties [19]. This compound displays anti-proliferative effects on cancer cells and has been investigated for its potential in preventing tumor growth. Additionally, in the field of neurology, ferulic acid has been explored for its neuroprotective capabilities, including its ability to mitigate neuronal damage and inflammation. We explored the synergistic potential of luteolin and ferulic acid in inhibiting GBM, recognizing the urgent need for novel therapeutic approaches to manage this challenging malignancy. Our findings indicate that the combination of these two compounds exhibited a remarkable inhibitory effect on GBM. This synergistic interaction holds promise for circumventing therapeutic resistance commonly observed in GBM treatments. By harnessing the potential synergy between luteolin and ferulic acid, our research underscores the prospects of a transformative paradigm in oncological interventions, aiming to usher in a new era of effective GBM therapy.

**Fig 1:**
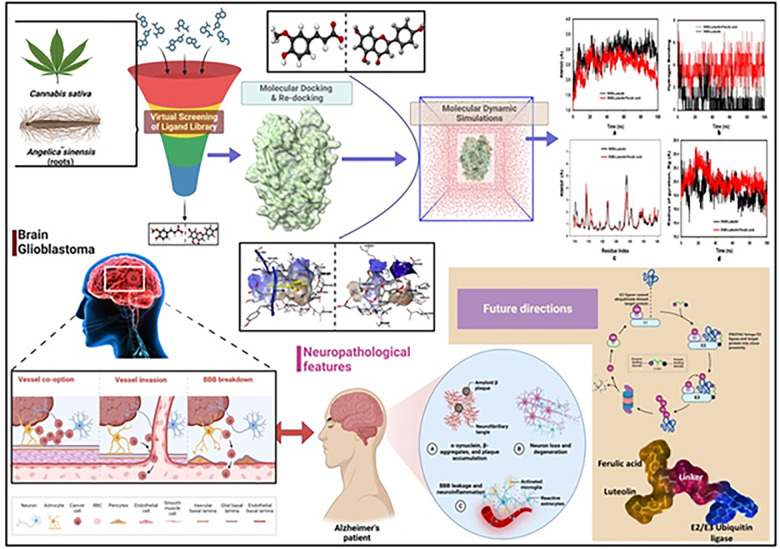
Workflow of the active inhibition using luteolin and ferulic acid in GBM and other neurodiseases and the future prospects (Created with Adobe Illustrator and BioRender.com).

In the rapidly evolving landscape of neuro-oncology and neurodegenerative research, our study offers an unprecedented trajectory that harmonizes the intricate biological mechanisms underlying GBM and AD. Specifically, we have deepened the understanding of the confluence between Aβ peptide accumulation in glioma cells and its potential relevance to neurodegenerative conditions such as AD. Our research is built on a robust analytical framework that dives into the most granular aspects of molecular biology. Utilizing an array of advanced computational methodologies including machine learning algorithms and data analytics, we carried out a comprehensive evaluation of the PubChem database. This exhaustive investigation led us to identify a novel chemical entity with a high degree of specificity against PTPRZ, a member of protein tyrosine phosphatase (PTP) family, a key protein implicated in GBM pathology [20]. The computational techniques employed in this study were carefully refined to maximize accuracy. Structure-based docking and virtual screening were synergistically integrated to enable the identification of a compound with unparalleled inhibitory efficacy against PTPRZ. Subsequent molecular dynamics simulations confirmed our findings, corroborating the structural stability and functional effectiveness of the identified chemical entity. A critical facet of our research was the exploration of the synergistic relationship between luteolin and ferulic acid, which are two naturally occurring compounds. These compounds were found to interact in a manner that amplified their individual therapeutic properties, thus accentuating their collective potential as a treatment modality in the context of both GBM and AD (Fig 1). In summary, our study serves as a cornerstone in not only forging a meaningful connection between two distinct neurological spectra but also in enriching the arsenal of neuro-oncological interventions. The chemical entity we identified, unearthed through state-of-the-art computational techniques, heralds a monumental shift in our therapeutic approach to both GBM and AD. It significantly extends the frontiers of current medical research, promising to redefine treatment protocols in these complex and challenging domains.

## 2. Materials and methods

### 2.1. Retrieval of protein and ligand structures

Our primary protein of interest was derived from the RCSB Protein Data Bank (PDB), specifically from the identifier RCSB Pdb ID: 5H08 (PTPRZ) [20]. To decode the intricate mechanisms underlying the binding of the glio-protein, three-dimensional structures in SDF format pertaining to ligands were extracted from the PubChem database. The molecules underwent energy refinement using the UCSF Chimera software [21] comprising a 900-step Conjugate Gradient method and a subsequent 1000-step steepest-descent optimization. These refined structures were then transformed into pdb format using Open Babel. Once the Gasteiger charges were integrated, we incorporated the AMBER ffSB14 force field to fine-tune the partial charges [22].

### 2.2. Compound assessment & selection

The integrity of the bond formed between an enzyme’s active region and its substrate is of utmost importance, as underscored by previous studies [20, 23]. The 2022 edition of BIOVIA Discovery Studio Visualizer assisted in the successful tethering of our selected compound to the active site of the protein, aiming for optimal binding tenacity. The protein complex’ receptor grid blueprint was devised using AutoDock Vina [24, 25], which subsequently enabled the evaluation of a set of 10 compounds. The phytochemical showing the apex of binding energy to the macromolecule was earmarked for in-depth scrutiny.

### 2.3. Enhanced docking protocols

Upon completion of the virtual screening phase, luteolin and ferulic acid emerged as the compounds to be profiled for receptor grid design using AutoDock MGL v1.5.6. Command-line tasks were managed efficiently by the Vina Wizard [26, 27]. Docked conformations, stored as pdbqt, were dissected for insight using PyMol and Discovery Studio Visualizer (2021 version). Before progressing to the main docking sequences, the target protein was subjected to steepest descent calibration, entailing 1000 iterations and the incorporation of the AMBER ff4 force field. Subsequent to the hydrogen amalgamation, the parameters were established at X = 45.42 Å, Y = 38.78 Å, and Z = 60.24 Å with a grid spacing of 1.25 Å. The Lamarckian Genetic Algorithm was employed to appraise protein-ligand complexes using their binding free energy (ΔG) determinants [28, 29].

### 2.4. Molecular dynamic simulations

MD simulation probes were initiated on the crystalline configurations of PTPRZ when allied with luteolin alone and in combination with ferulic acid, utilizing the Desmond 2020.1 suite by Schrödinger, LLC [30–32]. All simulations, including control setups, maintained a consistent temperature setting of 27°C. The computational framework harnessed the prowess of the OPLS-1005 force field [33, 34] and an explicit solvent paradigm with SPC water molecules encapsulated within a defined salvation boundary. To neutralize system charge, Na^+^ ions were introduced, followed by the addition of a 0.15 M NaCl solution to mimic physiological conditions. The system underwent initial equilibration in the NVT ensemble, succeeded by a phase in the NPT ensemble [35]. Throughout these dynamical computations, the Nose-Hoover chain coupling mechanism was upheld [36]. The particle mesh Ewald technique was used to elucidate extensive electrostatic interactions. Each trajectory was computed using the RESPA integrator, culminating in a comprehensive production run lasting 100 ns [37, 38].

## 3. Results

### 3.1. Synergistic docking insights: Luteolin and ferulic acid interaction with PTPRZ

**Fig 2:**
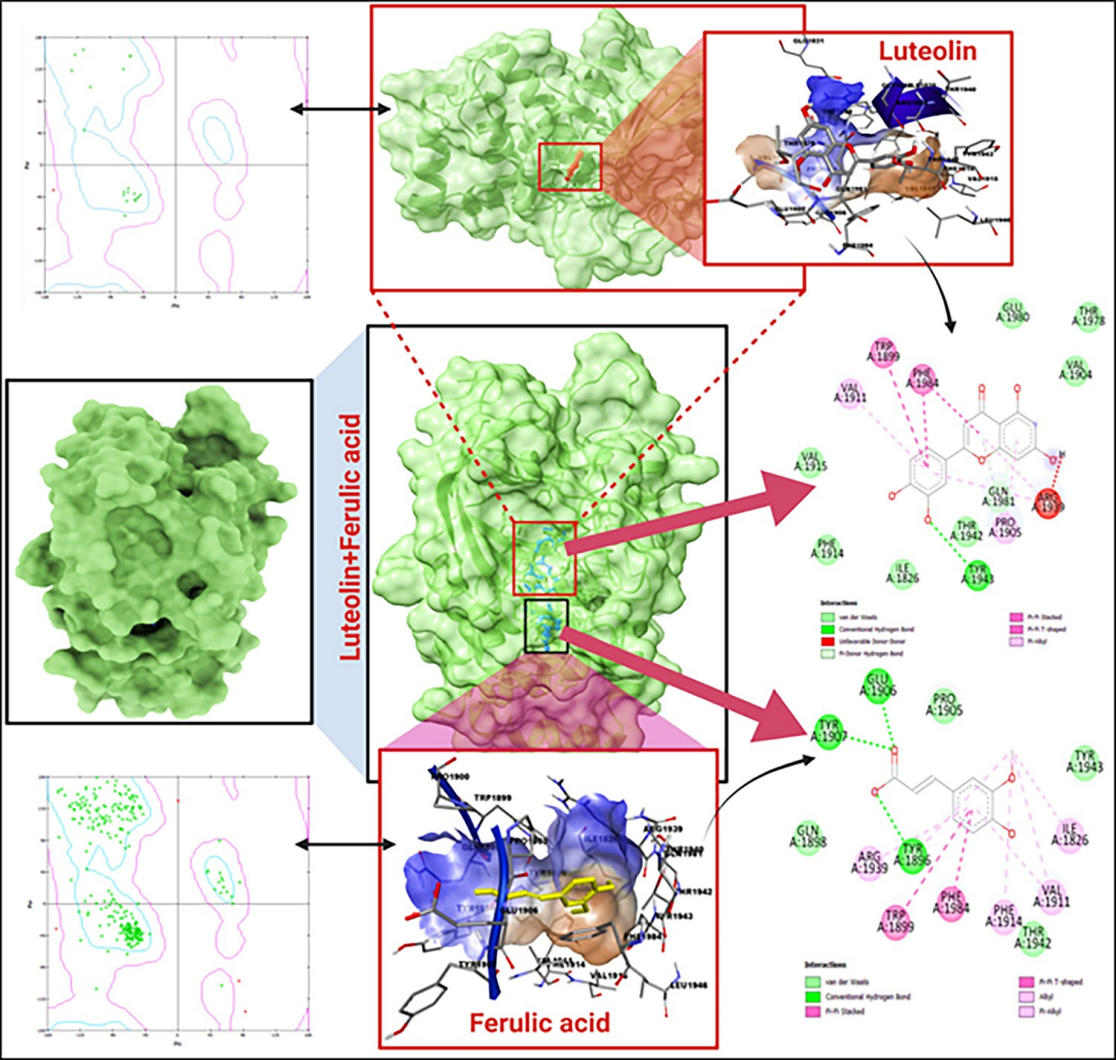
Molecular docking of 5H08 (PTPRZ) + (Luteolin + Ferulic acid); Ramachandran plots at left panel and the 2-D interaction diagram on right panel.

In the comprehensive study of protein-ligand interactions, a cardinal principle is that ligands with diminished binding energy scores typically exhibit heightened affinities to their corresponding target proteins. Our systematic investigation embarked with an initial focus on the docking process involving luteolin. This particular phase yielded a binding energy score of -9.6 kcal/mol. Intriguingly, when we proceeded with ferulic acid for a subsequent docking experiment, the binding score saw an augmentation to -10.5 kcal/mol. This enhancement is not merely numerical; it points towards a probable synergistic effect engendered by the concurrent interaction of the two ligands. To attain a nuanced understanding and to ensure the accuracy of our findings, we deployed advanced computational tools, namely the AutoDock Vina wizard and PyRx. These tools facilitated the docking of 10 distinct phytocompounds, all possessing unique three-dimensional structures, with our target protein. Their binding affinities, which serve as pivotal metrics in this domain, are meticulously cataloged in Table 1. Of particular significance was our observation during the redocking phase. The composite ligand, luteolin combined with ferulic acid, revealed a distinct and specialized binding pocket. This intricate interaction culminated in the ligands’ binding at the core pocket of the PTPRZ. The culmination of these experiments and analyses showed an impressive binding free energy of -10.5 kcal/mol, which is comprehensively illustrated in Fig 2. These findings underscore the potential of a synergistic approach to enhance molecular binding outcomes.

**Table 1:** A list of 10 phytocompounds.

Sl. No.	Phytocompounds	Synergistically docked Phyto active compounds	Binding energy (Kcal/mol)
1.	CID_92158	CID_100332	-4.6
2.	CID_ 472398529	CID_192158	-4.9
3.	CID_ 472396965	CID_119034	-7.1
4.	CID_ 135360080	CID_15559069	-4.9
5.	CID_49898702	CID_241572	-8.0
**6.**	**CID_ 5280445 (Luteolin)**	**CID_445858 (Ferulic acid)**	**-10.5**
7.	CID_482038412	CID_3981577	-7.9
8.	CID_472388395	CID_5280343	-6.5
9.	CID_482038413	CID_5280443	-7.9
10.	CID_241570	CID_5280445	-5.1

### 3.2. In-depth molecular dynamics examination of synergistic interaction between luteolin, ferulic acid, and the PTPRZ

Our extensive molecular dynamics (MD) simulation, which spanned over 100 ns, served as a focused lens for the intricate interactions between luteolin, ferulic acid, and PTPRZ. An essential cornerstone of this exploration is the dual-docking strategy. The initial phase involved docking with luteolin, which resulted in a binding score of -9.6 kcal/mol. Subsequently, a redocking endeavor was undertaken with ferulic acid, which remarkably enhanced this score to -10.5 kcal/mol. This shift in binding energy revealed the synergistic binding effect of luteolin and ferulic acid combination. Diving deeper into the results:

#### 3.2.1. Root mean square deviation (RMSD)

**Fig 3:**
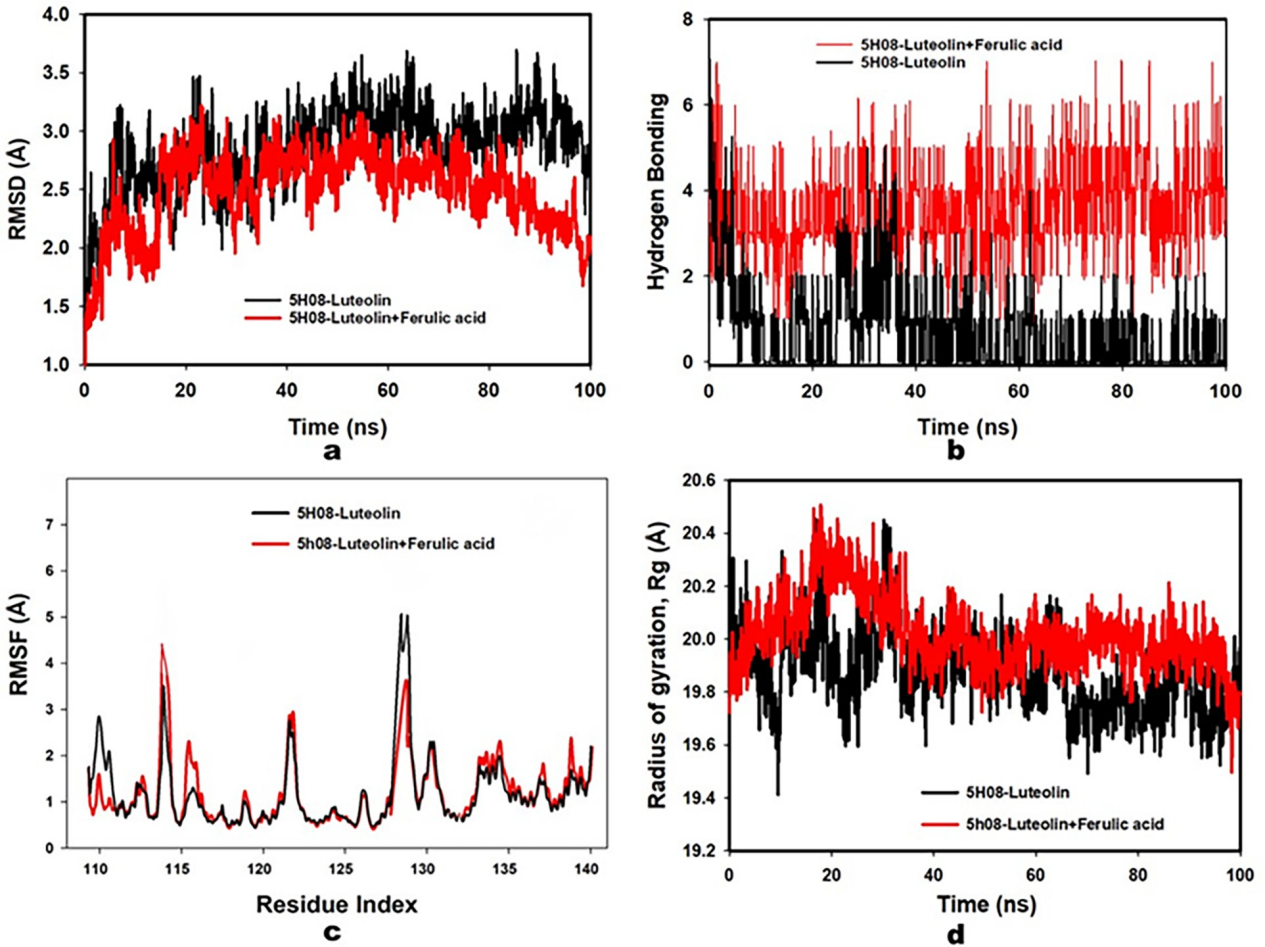
**(a)** RMSD of 5H08 (PTPRZ) + (Luteolin + Ferulic acid) after 100 ns run; **(b)** RMSF of 5H08 (PTPRZ) + (Luteolin + Ferulic acid) after 100 ns run; **(c)** Radius of Gyration of 5H08 (PTPRZ) + (Luteolin + Ferulic acid) after 100 ns run; **(d)** Hydrogen bonding of 5H08 (PTPRZ) + (Luteolin + Ferulic acid) after 100 ns run.

A compelling metric of stability emerged when examining the C-alpha backbone of the PTPRZ-ligand complex with a RMSD value of only 0.56 Å. While luteolin + ferulic acid presented an early-stage deviation, by the culmination of the 100 ns period, it demonstrated a firm, stabilized trajectory, as illustrated in Fig 3A.

#### 3.2.2. Root mean square fluctuations (RMSF)

The C-alpha backbone of PTPRZ stood out as a beacon of structural consistency. The encapsulated amino acids within demonstrated minor fluctuations, affirming their resilience throughout the simulation’s duration. After exhaustive simulation, a side-by-side comparison of the terminal conformation of PTPRZ against its initial reference resulted in marked variations, especially within the residue bracket of 390–470 (Visualized in Fig 3B).

#### 3.2.3. Hydrogen bond dynamics

A consistent theme throughout the 100 ns interval was hydrogen bond dynamics. Between ferulic acid and PTPRZ, an enduring presence of three bonds was observed, reiterating the steadfast nature of their interaction, and hinting at the mutual synergistic influence of luteolin and ferulic acid in maintaining such bonds (Fig 3C).

#### 3.2.4. Radius of gyration (Rg) dynamics

Serving as an index of the conformational robustness of the ligand-bound protein, Rg values manifested a steady pattern, floating between 19.6 and 19.8 Å. The unwavering nature of these values is illustrated in Fig 3D, attests to the structural integrity and compactness of the complex throughout the investigative process.

Comprehensive MD simulation accentuates the robust and synergistic interactions between PTPRZ, luteolin, and ferulic acid. The array of metrics and methodologies employed in this investigative journey provides unequivocal evidence of the structural fidelity and dynamic equilibrium of the resultant complex, particularly underscoring the synergistic effects of luteolin and ferulic acid.

### 3.3. Protein-ligand interactions & ligand torsion profile

**Fig 4:**
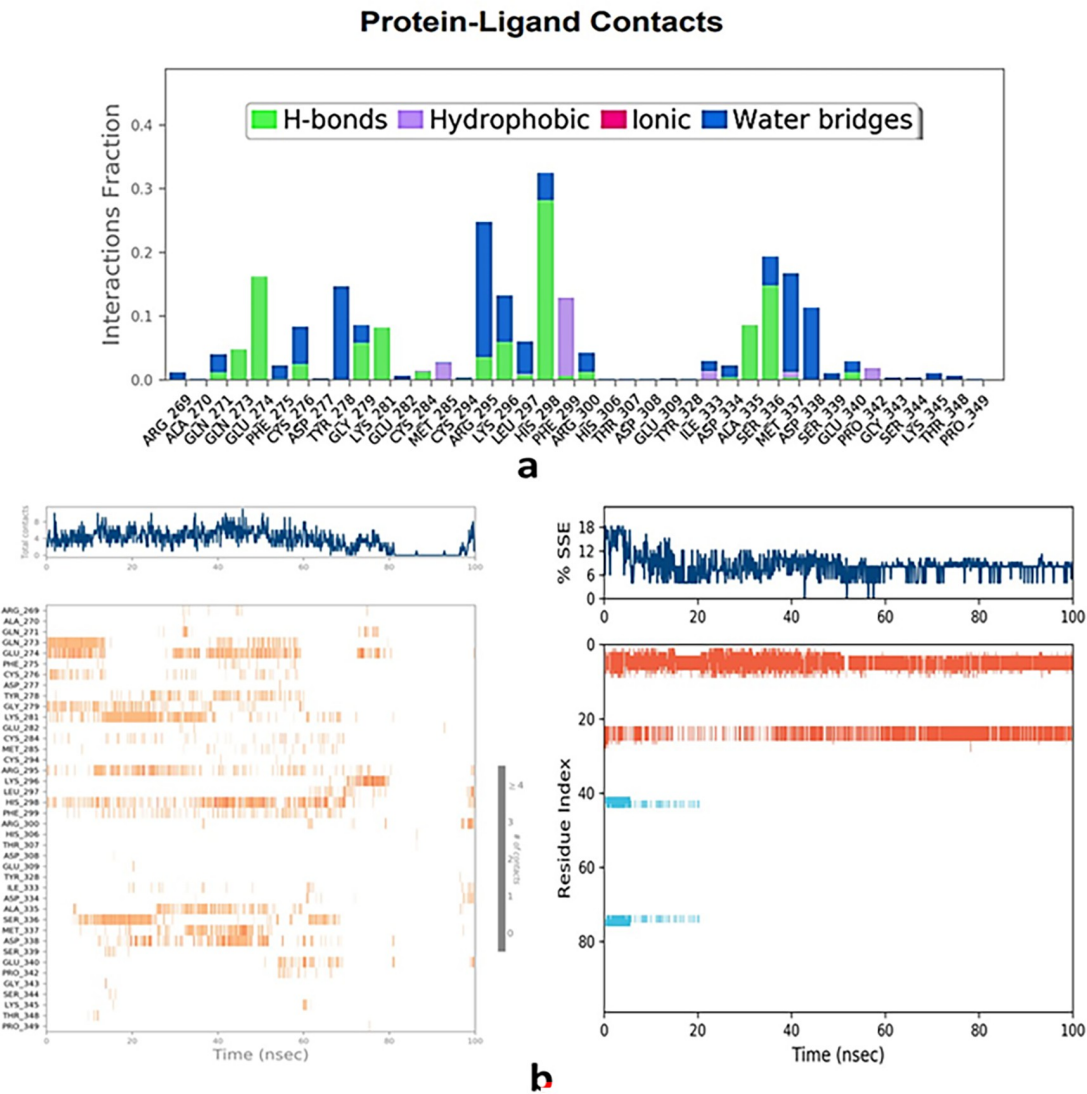
Types of bonds formed in 5H08 (PTPRZ) + (Luteolin + Ferulic acid) complex and contacts100 ns run.

**Fig 5:**
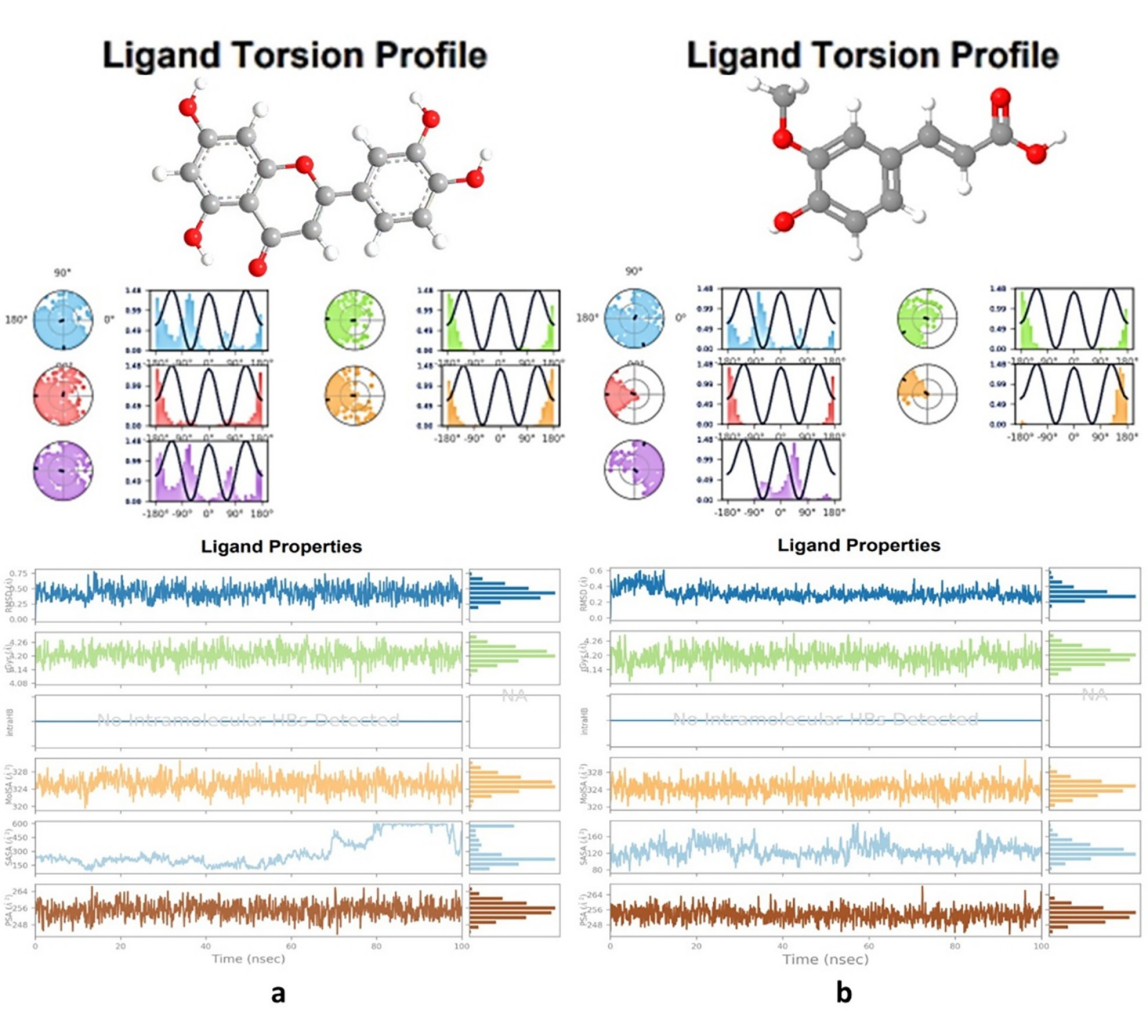
Torsion profiles of luteolin & ferulic acid after 100 ns run.

The intricate ligand interactions of the combined luteolin and ferulic acid with the projected docked residues of PTPRZ showed a robust establishment of hydrogen bonds. Additionally, there were notable non-bonded interactions encompassing hydrophobic interactions and water bridge formation (Figs 4 and 5). The synergy between luteolin and ferulic acid was pivotal in mediating these interactions, ultimately contributing to the enhanced stability of the protein-ligand complex.

## 4. Discussion

In the annals of medical history, the role of phytoconstituents as potent pharmacological agents against a broad spectrum of diseases remains indelible. Particularly noteworthy is their potency against neurodegenerative diseases such as Alzheimer’s disease and complex neuro-oncological disorders such as glioblastoma. The complexities inherent to Alzheimer’s, when explored in parallel with the intricate mechanisms driving glioma-genesis, present a fascinating mosaic of potential intersections. Although the clinical manifestations and pathological trajectories of Alzheimer’s disease and glioblastoma are distinct, an exploration of their overlapping molecular signatures could potentially inaugurate transformative therapeutic avenues. The current study exemplifies a seminal endeavour in this direction, employing a meticulous approach to decode the molecular intricacies of glioblastoma, while concurrently identifying overlaps with Alzheimer’s pathology. To achieve this, we harnessed the capabilities of contemporary proteomic methodologies, enabling us to delineate both shared and unique markers of therapeutic resistance pertinent to these disorders. Marrying the therapeutic efficacy of natural compounds with the precision and scalability afforded by computer-aided drug design, our methodological framework aimed to define the future of targeted therapies.

Central to our inquiry was the elucidation of the synergistic effects of luteolin and ferulic acid. These compounds were rigorously evaluated for their interactions with receptor-type protein tyrosine phosphatases enzymes implicated in the etiology of both glioblastoma and certain neurodegenerative conditions. Our results, validated through advanced molecular docking techniques and corroborated by molecular dynamics simulations, highlight the powerful therapeutic potential of this compound, particularly in the context of glioblastoma. Although luteolin and ferulic acid demonstrated significant anti-glioblastoma capabilities, their relevance in Alzheimer’s disease pathology also surfaced as a noteworthy finding. Although our computational research offers compelling insights, it is prudent to acknowledge the necessity for rigorous *in vivo* and preclinical validation. While other phytoconstituents, notably those derived from *Angelica sinensis* and *Cannabis sativa*, exhibited anti-tumor attributes, the coordinated inhibitory dynamics of luteolin and ferulic acid took precedence, specifically regarding their binding energies in drug-target engagements.

In conclusion, the current investigation underscores the pharmacological properties of luteolin and ferulic acid, advocating their immediate consideration as leading candidates for novel PTPRZ inhibitors. This can potentially revolutionize therapeutics in the realm of glioblastoma while simultaneously offering new investigative pathways for Alzheimer’s research. Thus, our findings serve as a critical touchstone, championing an integrative approach in the relentless quest for efficacious therapies in the treatment of both neuro-oncological and neurodegenerative disorders.

## 5. Conclusions

In the intricate realm of neuroscientific endeavors, our study emerges as a beacon, elucidating the conjoint therapeutic prowess of luteolin and ferulic acid against the multifaceted challenges posed by GBM and AD. By seamlessly melding advanced proteomics and state-of-the-art computer-aided drug design paradigms, we have delved profoundly into the molecular nuances of these phytoconstituents, aiming to discern their harmonized potential. While the individual merits of luteolin and ferulic acid have been extensively documented in the scientific literature, it is their synergistic interplay borne out of a meticulous orchestration of molecular interactions that unveils an unprecedented therapeutic frontier against the conjoined pathways evident in glioblastoma and AD. This exploration bridges the diverse terrains of neurology and oncology, providing a holistic vantage point for nuanced molecular choreography governing these debilitating ailments. Although our *in-silico* findings show an optimistic trajectory, the transition from computational insights to tangible clinical applications necessitates stringent empirical validation. The compelling data emerging from our study mandates rigorous *in vivo* evaluations, ensuring that luteolin-ferulic acid synergy translates into clinically viable interventions. Our study amplifies the collective capabilities of luteolin and ferulic acid, postulating their potential synergistic supremacy over their individual attributes. This confluence of knowledge not only deepens our comprehension but also catalyzes the development of novel treatments for formidable challenges such as glioblastoma and Alzheimer’s disease.

Moreover, the nexus between traditional botanical knowledge and state-of-the-art technological advancements emphasizes the importance of collaborative research. By merging these realms, we can more adeptly harness the therapeutic potential of phytoconstituents such as luteolin and ferulic acid. Proteolysis targeting chimeras (PROTACs), given their groundbreaking protein degradation mechanism, further accentuate this potential, offering a multifaceted approach to address complex, dysregulated pathways [39–42].

## Supporting information

S1 File (XLSX)
